# Semiclosure wound therapy plus negative pressure wound therapy for an older patient with grade 4 diabetic foot with concomitant vascular occlusion: A case report

**DOI:** 10.1097/MD.0000000000017786

**Published:** 2019-11-01

**Authors:** Yafeng Yan, Wenfeng Li, Yan Song, Pei Yin, Zongze He, Yanping Gong, Lili Peng

**Affiliations:** a Department of Geriatric Endocrinology, The Second Medical Center, The Chinese People's Liberation Army General Hospital; b National Clinical Research Center for Geriatric Diseases; c Department of Orthopedic; d Department of Nephrology, The Fourth Medical Center, The Chinese People's Liberation Army General Hospital, Beijing, China.

**Keywords:** grade 4 diabetic foot, infection, negative pressure wound therapy, older patient, semiclosure of the wound

## Abstract

**Rationale:**

Grade 4 diabetic foot (DF) is a severe infection that causes bone destruction, osteomyelitis, and osteoarticular damage, which, in turn, can lead to serious dry or wet gangrene, or amputation. DF is extremely difficult to treat.

**Patient concerns:**

A 71-year-old female patient with long-term diabetes complicated with uremia, who undergoes regular hemodialysis 2 to 3 times per week, was admitted with grade 4 DF with *Pseudomonas aeruginosa* infection, and concomitant vascular occlusion of the lower extremities. The patient had a concurrent nutrition and electrolyte disorder.

**Diagnoses:**

The patient was diagnosed with type 2 diabetes, grade 4 DF, postamputation of the 2nd toe, vascular occlusion of the lower extremities, atherosclerosis, uremia, hypoproteinemia, and electrolyte disturbances.

**Interventions:**

Treatment with antibiotics and comprehensive measures aimed at improving nutrition and microcirculation, controlling blood glucose, as well as balancing electrolytes were performed to ameliorate the general conditions. Nibbled debridement was used to remove devitalized tissues each time to maintain as much vital cells as possible. Open therapy was used for necrotic tissues, and dressings therapy was used simultaneously for the infected lesion. This combined treatment, involving open therapy with dressing, is referred to as “semiclosure wound therapy.” Negative pressure wound therapy (NPWT) was used after a fistula formed.

**Outcomes:**

During the treatment procedure, the gangrene 3rd toe was spontaneously shed; the necrotic 1st toe was removed by surgery. The wound gradually healed after 3 months of open therapy combined with dressing. High location amputation was avoided.

**Lessons:**

Semiclosure, which constitutes open therapy combined with the use of dressings, plus NPWT can preserve vital skin cells in the wound and control the aggravation of the infection. It is an effective and novel measure that prevents DF amputation in old patient and promotes wound union.

## 1 Introduction

Diabetic foot (DF) is one of the major complications of diabetes mellitus that mainly occurs due to neuropathy, and minor vascular lesions or microcirculation disorders of the lower limbs. In these pathological contexts, minor injury or infection can generate a skin ulcer and local tissue necrosis, or, in extreme cases, dry and wet gangrene. These changes occur partly due to pre-existing foot abnormalities, such as numbness and loss of sensitivity to temperature or pain. DF has high incidence, high disability rate, and is associated with significant medical costs. According to estimates by an international working group on lower limb amputation, globally, 25% to 90% of amputations are related to diabetes mellitus.^[1]^ The number of patients with DF is continually increasing, and DF amputations account for 50% of nontraumatic lower extremity amputations worldwide.^[2]^ Moreover, studies have shown that amputation of one limb increases the risk of amputation of another limb; specifically, 50% of patients undergoing amputation due to DF require another amputation within 5 years.^[3]^ Research estimates lifetime incidence of foot ulcers among patients with diabetes at around 15%, although some studies indicate it may be as high as 25%.^[4]^ In developed countries, 5% of patients with diabetes develop DF, leading to diminished quality of life and high healthcare costs. In developing countries, the incidence of DF is estimated to account for 40% of medical expenses related to diabetes mellitus.^[5]^

Foot ulcers include arterial ulcers, venous ulcers, and mixed-type (arterial and venous) ulcers. Arterial ulcer is a term commonly used to refer to ischemic ulcers. Such impairment can occur acutely (eg, trauma, thrombosis) or chronically (eg, atherosclerosis). Venous ulcer, also known as stasis ulcer, is the most common etiology of lower extremity ulceration. It is important for clinicians to be aware of the different types of ulceration and their treatment modalities. Thus, an appropriate wound therapy is crucial for patient with DF, especially old patient.

## 2 Case report

### 2.1 Clinical data

A 71-year-old woman was admitted to our department with infected wounds that had been progressing for 3 months. In July 2016, the patient fell at home and injured the skin of the left calf. Subsequently, scabs formed within a week, while the 2nd toe on the left foot gradually darkened and turned tender over the next 2 months. In September, left femoral artery angiography plus balloon dilatation were performed at a local hospital, because of arteriosclerotic stenosis of the lower extremities. During the same month, amputation of the 2nd toe on the left foot was performed. Despite the amputation, the patient's condition continued to deteriorate. Finally, on October 23, 2017, the patient was admitted to our department.

The patient's medical history included 25 years of diabetes poorly controlled with insulin and treated with oral hypoglycemia medicine. She also had an 18-year history of uremia, for which she underwent regular hemodialysis 2 to 3 times per week, and 10 years of atherosclerosis of the lower extremities.

Physical examination revealed rupture, redness, swelling, exudation, and darkening of the area surrounding the infected wound. The wound reached into the bone and it involved 1st, 2nd, and 3rd toe of the left foot and two-thirds of the plantar area, which was covered with dark scabs and yellow-green purulent secretions. The wound area was about 9 cm × 5 cm, and the whole foot was swollen (Fig. 2). The undulation of the foot dorsal artery disappeared, in addition to plantar bone defect and necrosis. Blood test showed hypoproteinemia (plasma albumin of 24.3 g/L), electrolyte disturbances (hyponatremia, hypokalemia, and hypochloremia), fasting blood glucose of 14.5 mmol/L, and postprandial blood glucose of 15.8 mmol/L.

**Figure 1:**
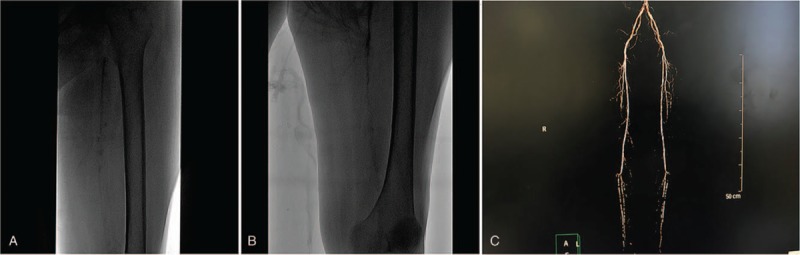
Diabetic foot (DF) condition after admission.

Considering serious foot infection and toe necrosis, and vascular occlusion of the lower extremities (Fig. 1), colleagues from Orthopedics and Traumatology Department recommended amputation of the leg above the knee. Nevertheless, the patient's age, serious malnutrition, uremia, 18 years of hemodialysis, electrolyte imbalance, and potential cardiac and lung insufficiency made the operation high-risk. Following a consultation, the family of the patient declined their consent for the surgery and asked for conservative therapy instead. In response, the patient was at first treated with adequate nutrition therapy, electrolyte infusion, and measures to improve circulation. Further, a systemic wound semiclosure strategy was adopted as DF wound therapy to control infection, promote tissue regrowth, and favor a full union of the wound edges.

**Figure 2:**
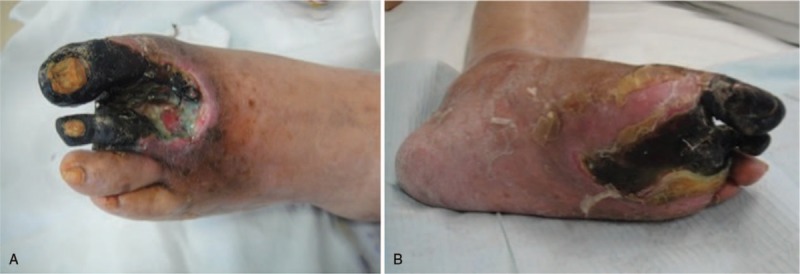
Vascular occlusion images of the lower left extremity in the diabetic foot (DF) patient.

### 2.2 Treatment

#### 2.2.1 Semiclosure wound therapy and with antiinfection treatment

Immediately after the patient was admitted, a wound specimen was collected, and drug-sensitivity test for bacteria was conducted. Three days after admission, the wound specimen culture revealed infection with *Pseudomonas aeruginosa* and *Enterococcus faecium*. Consequently, the patient received an intravenous injection of 3.0 g Sulperazon (Pfizer, China) once daily.

Nibbled debridement therapy was performed, which involved clearing a smaller patch of necrotic tissue with higher frequency (usually once per 2–3 days, while once per 5–7 days during negative pressure wound therapy [NPWT]) than in traditional therapy. This approach allowed to continually evaluate clearing progress, avoid normal tissue damage, prevent bleeding, and preserve as much vital skin cells as possible. This, in turn, helped induce growth of fresh tissues in the wound of foot dorsum.

**Figure 3:**
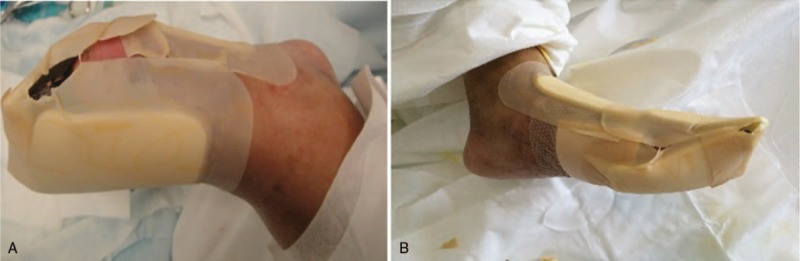
Semiclosed treatment of the infected wound. (A) Dorsum and (B) the plantar.

Open therapy is usually applied to an infected wound. In this patient, silver ion antibacterial dressing (Comfeel silver ion hydrocolloid yarn, Coloplast, Denmark) was applied at wound site to restrain wound infection. Simultaneously, nonsticky foam dressing (Biatain Foam Dressings) was used as the outer layer as it is high-absorbent and has not been contraindicated for infected wound closure (Fig. 3). This treatment method, involving a combination of open therapy with dressing, is referred to as “semiclosure wound therapy” (Fig. 3).

#### 2.2.2 Spontaneous shedding of the 3rd toe with dry gangrene

**Figure 4:**
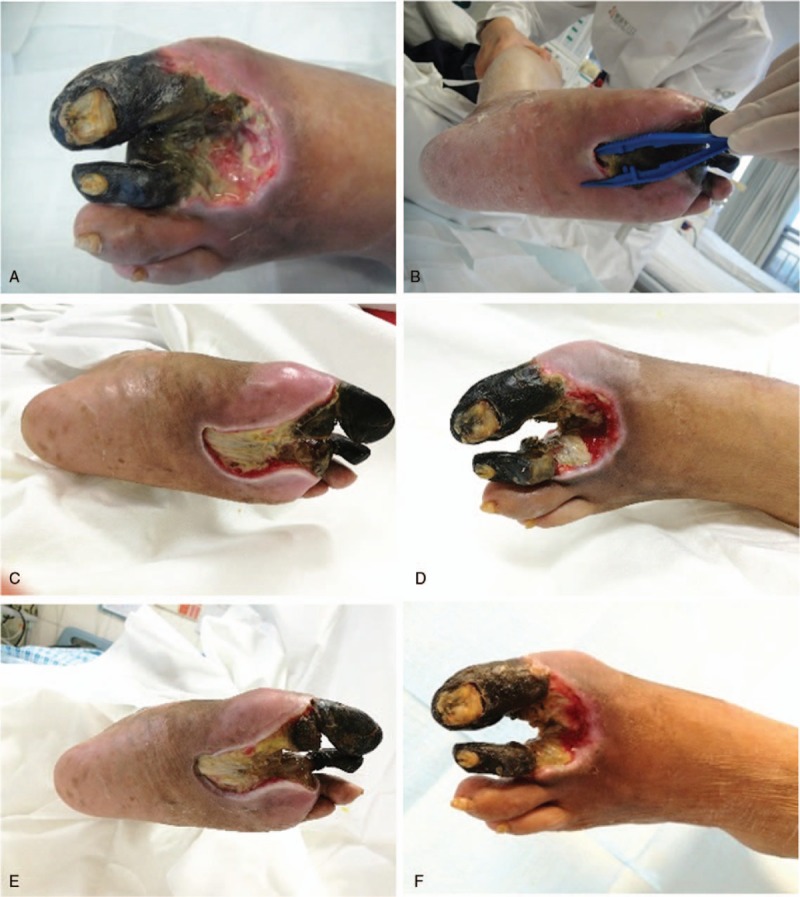
Diabetic foot (DF) condition at 2 days (A, B), 19 days (C, D), and 23 days posttherapy (E, F).

Spontaneous shedding of the 3rd toe with dry gangrene occurred on the 19th day of hospitalization during dressing change (Fig. 4). At this stage, following progressive removal of black scabs, exudation, and necrotic tissues, surviving skin cells of this DF patient were protected to the most extent.

#### 2.2.3 Excision operation and continuous semiclosure wound therapy

**Figure 5:**
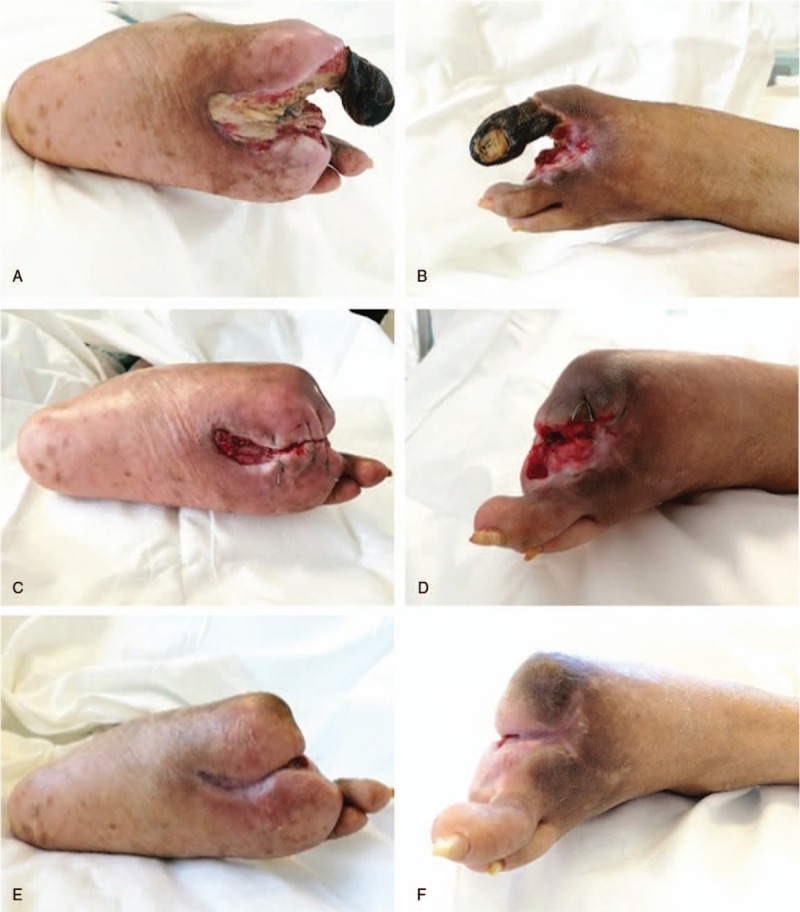
Diabetic foot (DF) condition at 56 days, 71 days, and 90 days posttherapy. (A, B) The dorsum and the plantar at 56 days posttherapy. Spontaneous shedding of the 3rd toe with dry gangrene. (C, D) Resection of the necrotic foot thumb at 56 days posttherapy. (E, F) The dorsum and sole of the foot at 71 days posttherapy. (G, H) The dorsum and sole of the foot at 3 months posttherapy.

After the spontaneous shedding of the 3rd toe, another assessment of the foot wound was conducted. Subsequently, the necrotic 1st toe and fascia in the plantar wound were removed. Slack suture was performed at the plantar wound to prevent excessive tension; normal docking suture was used at the back of the thumb excision position (Fig. 5). Fibroblast growth factor (ShuangLu Pharmaceutical Liquid Co., LTD. Beijing) was administered to the wound for 3 weeks.

#### 2.2.4 Formation of wound fistula and negative pressure wound therapy

**Figure 6:**
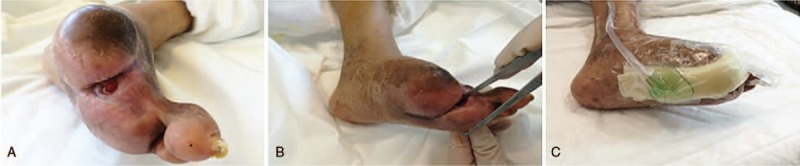
Negative pressure therapy and semiclosed treatment. (A, B) Fistula formation and (C) negative pressure therapy.

**Figure 7:**
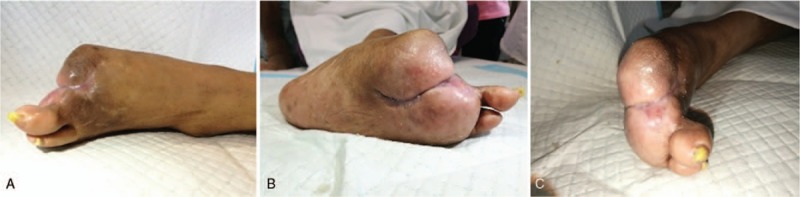
Outcome of this diabetic foot (DF) patient. (A) Dorsum, (B) plantar, and (C) fingers.

Following systematic wound therapy and nursing care, although the DF ulcer gradually healed, a fistula 2 cm in length emerged, with the estimated wound area of 1.5 cm × 1 cm (Fig. 6 A, B). NPWT, which could promote rapid growth of the granulation tissue in the fistula, was adopted. Considering the fragility of the patient's skin and arteriosclerotic stenosis of the lower extremities, negative pressure was set at a maintenance level with low pressure to prevent secondary damage. Meanwhile, when adjuvant medications to promote proliferation of vascular cells and skin cells were administered, the negative pressure drainage tube was clamped for 20 to 30 minutes to promote adequate access and absorbance of drugs to the wound (Fig. 6C). Ultimately, after 90 days of treatment (including 3 weeks use of NPWT and 3 weeks of antibiotics given), the foot lesions healed, and the fistula closed. No recurrence was detected during the next 6 months follow-up. High position amputation was successfully avoided, and our therapy greatly improved the patient's quality of life (Fig. 7).

## 3 Discussion

As diabetes prevalence increases, the rates of DF rates and related amputations have also been increasing. The Wagner classification is a DF classification method proposed in 1976,^[6]^ whereby the severity of a DF wound is divided into 6 grades based on the depth of the ulcer.

- Grade 0: high risk of foot ulcer, currently no ulcer.
- Grade 1: Superficial diabetic ulcer.
- Grade 2: Ulcer extension involves ligament, tendon, joint capsule or fascia, without abscess or bone infection.
- Grade 3: deep ulcer with abscess or osteomyelitis.
- Grade 4: Gangrene to portion of forefoot.
- Grade 5: Extensive gangrene of foot.

Our patient was classified as Wagner grade 4 DF. Concurrent arteriosclerotic stenosis of the lower extremities, in addition to prolonged wound infection, and multiple organ dysfunction were risk factors associated with wound nonunion, toe amputation, and even high position amputation. The success of this patient's treatment depended on open therapy combined with dressings, and the concurrent use of dynamic complementary NPWT methods.

This patient had a mixed-type (arterial and venous) ulcer. It is widely accepted now that the wound could be classed as black, red, and yellow, where each color represents a specific pathology. “Yellow” stands for more purulent secretion and exudate; as a result, drier dressings with good drainage and antiinfection effect are preferred, and oily or closed dressings should be avoided. “Black” wounds are dry wounds with or without surface necrotic scab shell; in such a case, an airtight dressing is better for skin softening and facilitating debridement.^[7,8]^ For a patient with a wound that is a combined “black” and “yellow” wound, open therapy combined with dressings is a novel and better choice. We have named this method “semiclosure wound healing.”

Proper debridement methods and appropriate wound healing techniques halted DF aggravation in this patient. Regular clinical debridement approaches include: surgical debridement, where conventional surgical instruments like scalpel and scissors are applied to clear the necrotic tissues, muscles, fascia, and bone chips from damaged bones; physical debridement, where the necrotic tissues and tissue debris are rinsed with water or scraped with instruments; autolysis debridement, where devitalized tissues are liquefied, softened, and removed by the endogenous lysozymes in the wound; enzymological debridement, in which exogenous enzymes are applied to decompose and scavenge necrotic or devitalized tissues; and biological debridement. The selection of debridement approaches depends on the patient and wound condition, such as presence of infection, extent of necrosis, vascular distribution of the wound, pain tolerance of the patient, the environment for wound management, and access to certain debridement approaches.^[9]^ Surgical debridement refers to removal of large amounts of necrotic tissue over a short period of time, which carries the risk of damaging a portion of vital tissues and cells. Nibbled debridement is an improved version of surgical debridement, whereby the process is divided into stages, and a section of devitalized tissue is removed at a time.^[10]^ Overtime, the wound becomes entirely filled with fresh tissue. Dynamic determination of precision debridement methods played an important role in the present patient's healing.

Previous studies have shown that, when the cause of infection is confirmed, topical antibacterial agents and systemic antibiotics should be applied to the wound.^[11]^ In the current study, silver ion antibacterial dressing was applied at the infection phase. Silver ion dressing is a type of spectral antiseptic dressing made from hydrophilic particles and hydrophobic polymers, which continuously releases low concentration of silver ions, which damage bacterial RNA and DNA, and effectively suppress microbial growth.

When a fistula formed, a vacuum-assisted wound closure was used. Vacuum-assisted closure (VAC) is a recently developed technique, which falls under the category of NPWT.^[12,13]^ It utilizes an intelligent control vacuum device to build a closed environment surrounding the wound via connective pipes and filling dressings. VAC generates negative pressure for the local wound area at an intermittent or a sustained pattern, aiming to raise tissue blood supply, diminish tissue edema, and promote wound healing and union.^[14,15]^ When the wound is deep, and the infection reaches deep tendon and loose connective tissue, it is difficult to drain, because the edema occurs due to the influence of cytokines, chemokines, peptides, and prostaglandins. Under such conditions, it is important to change passive drainage to active drainage, such as drainage by VAC, which could guarantee effective drain and promote healing.^[16,17]^ In the present case, negative pressure therapy was applied for 3 weeks, and the fistula wound eventually fully healed.

Furthermore, patients with diabetes, especially older-age patients, often have multiple complications or concomitant diseases. Thus, the initiation, development, and healing of older patients with DF are closely associated with blood glucose levels, electrolytes, nutrition, microcirculation, and other general conditions. Multidisciplinary and comprehensive management are essential. Fibroblast growth factor plays the auxiliary role in promoting repair and regeneration of the skin cells.

## 4 Conclusions

Wound care in older-age patients with severe grade 4 DF combined with vascular occlusion of the lower extremities is a great challenge for medical practitioners worldwide. The use of semiclosure of wound method could maximize vital cell growth, as well as effectively treat the infected wounds. The application of VAC in patients with fistula or deep infection provides a safe and effective measure to reduce the risk of DF amputation and, effectively improve the patient's quality of life.

## Author contributions

**Conceptualization:** Yafeng Yan, Wenfeng Li, Yan Song, Zongze He, Yanping Gong.

**Data curation:** Yan Song, Pei Yin, Zongze He.

**Formal analysis:** Yafeng Yan, Wenfeng Li, Yan Song, Zongze He.

**Investigation:** Yafeng Yan, Zongze He.

**Methodology:** Yafeng Yan, Wenfeng Li, Yan Song, Pei Yin, Zongze He, Yanping Gong.

**Project administration:** Yan Song.

**Resources:** Wenfeng Li.

**Supervision:** Yafeng Yan, Yanping Gong.

**Validation:** Yafeng Yan, Yanping Gong.

**Visualization:** Yafeng Yan.

**Writing – original draft:** Yafeng Yan.

**Writing – review & editing:** Wenfeng Li, Yan Song, Pei Yin, Zongze He, Yanping Gong, Lili Peng.
